# Effect of Hypoglycemia and Rebound Hyperglycemia on Proteomic Cardiovascular Risk Biomarkers

**DOI:** 10.3390/biomedicines12061137

**Published:** 2024-05-21

**Authors:** Manjula Nandakumar, Thozhukat Sathyapalan, Stephen L. Atkin, Alexandra E. Butler

**Affiliations:** 1 Research Department, Royal College of Surgeons in Ireland, Adliya P.O. Box 15503, Bahrain; mnandakumar@rcsi.com (M.N.); satkin@rcsi.com (S.L.A.); 2 Academic Endocrinology, Diabetes and Metabolism, Hull York Medical School, Hull HU6 7RU, UK; thozhukat.sathyapalan@hyms.ac.uk

**Keywords:** type 2 diabetes, hypoglycemia, glucose variability, cardiovascular markers

## Abstract

**Introduction**: Hypoglycemia has been associated with cardiovascular events, and glucose variability has been suggested to be associated with increased cardiovascular risk. Therefore, in this study, we examined the effect on proteomic cardiovascular risk protein markers of (i) mild iatrogenic hypoglycemia and (ii) severe iatrogenic hypoglycemia followed by rebound hyperglycemia. **Methods**: Two iatrogenic hypoglycemia studies were compared; firstly, mild hypoglycemia in 18 subjects (10 type 2 diabetes (T2D), 8 controls; blood glucose to 2.8 mmoL/L (50 mg/dL) for 1 h), and secondly, severe hypoglycemia in 46 subjects (23 T2D, 23 controls; blood glucose to <2.2 mmoL/L (<40 mg/dL) transiently followed by intravenous glucose reversal giving rebound hyperglycemia). A SOMAscan assay was used to measure 54 of the 92 cardiovascular protein biomarkers that reflect biomarkers involved in inflammation, cellular metabolic processes, cell adhesion, and immune response and complement activation. **Results**: Baseline to euglycemia showed no change in any of the proteins measured in the T2D cohort. With severe hypoglycemia, the study controls showed an increase in Angiopoietin 1 (ANGPT1) (*p* < 0.01) and Dickkopf-1 (DKK1) (*p* < 0.01), but no changes were seen with mild hypoglycemia. In both the mild and severe hypoglycemia studies, at the point of hypoglycemia, T2D subjects showed suppression of Brother of CDO (BOC) (*p* < 0.01). At 1 h post-hypoglycemia, the changes in ANGPT1, DKK1, and BOC had resolved, with no additional protein biomarker changes despite rebound hyperglycemia from 1.8 ± 0.1 to 12.2 ± 2.0 mmol/L. **Conclusions**: Proteomic biomarkers of cardiovascular disease showed changes at hypoglycemia that resolved within 1 h following the hypoglycemic event and with no changes following hyperglycemia rebound, suggesting that any cardiovascular risk increase is due to the hypoglycemia and not due to glucose fluctuation per se.

## 1. Introduction

An increased risk of cardiovascular disease is associated with type 2 diabetes [1], and the United Kingdom Prospective Diabetes Study (UKPDS) suggested that tight glycemic control reduced macrovascular complications [2], though subsequent studies did not [3,4,5]. With intensive glycemic control, all-cause mortality increased in the Action to Control Cardiovascular Risk in Diabetes (ACCORD) study [4], though the cause of the increased deaths remains unclear; however, this has been recently challenged with new modeling techniques, suggesting a protective effect of tight glycemic control [6]. Hypoglycemia is associated with platelet function changes through platelet hyperactivity due to sensitivity to prostacyclin 24 h following the hypoglycemic insult [7] in addition to hypoglycemia-induced platelet activation [8,9] mediated by elevation in adrenaline levels [10,11]. Induced hypoglycemia results in increased inflammatory and oxidative stress markers and metabolic changes in T2D subjects, and these markers remaining elevated at 24 h may explain some of these platelet-associated changes [12,13].

Glucose variability refers to changes in blood glucose levels occurring within minutes or hours, which can be particularly marked in diabetes [14]. It is recognized that hyperglycemia is associated with diabetes-related complications, which may be prevented by improved glycemic control [15]. Glucose variability has been suggested to be associated with increased cardiovascular risk [16], perhaps through increased oxidative stress [17], though a degree of controversy remains [18]. In a study of normal volunteers injected repeatedly with intravenous glucose to mimic intraday glucose variability (3 times 20 g of glucose intravenously over 5 min at intervals of one hour), acute effects on the cardiovascular risk protein markers bone morphogenetic protein 6 (BMP6); signaling lymphocyte activation molecular family 7 (SLAMF7); a disintegrin and metalloproteinase with a thrombospondin type 1 motif, member 13 (ADAMTS13); interleukin-1 receptor antagonist (IL1RA); pentraxin 3 (PTX3); interleukin 4 receptor alpha (IL-4RA); and lectin-like oxLDL (oxidized low-density lipoprotein) receptor 1 (LOX-1) were reported [19]. What is unclear is whether this is simply due to the increment in the blood glucose or whether it is the increment followed by the decrement that is responsible for the potential adverse cardiovascular risk marker profile and whether this response could be mimicked by a decrease in blood glucose alone. The aim of this study was to measure the panel of cardiovascular risk proteins previously reported for glucose variability [19] in type 2 diabetes (T2D) and control subjects undergoing iatrogenic-induced mild prolonged hypoglycemia versus more acute severe hypoglycemia that was reversed immediately with a glucose rebound leading to marked and rapid hyperglycemia.

## 2. Material and Methods

Two hypoglycemic study designs were employed using the same hypoglycemic clamp technique, and patient demographics are shown in Table 1. In the first mild hypoglycemic (mild-hypo) study design, a case-control prospective study in Caucasian adult (aged 40–53 years) patients with T2D (*n* = 10) and nondiabetic control (*n* = 7) subjects that achieved a blood glucose of 2.8 mmol/L (50 mg/dL), which was maintained for 1 h [12], with blood sampling at baseline and at the end of 1 h of hypoglycemia (prior to reversal); subjects did not have overt symptoms of hypoglycemia. (Ethical approval by Yorkshire and the Humber Research Ethics Committee, registered at www.clinicaltrials.gov (NCT02205996) and performed from November 2011–May 2013).

In the severe hypoglycemic (severe-hypo) study design, a case-control prospective study in Caucasian adult (aged 40–70 years) patients with T2D *(n* = 23) and nondiabetic control (*n* = 23) subjects that achieved a blood glucose of ≤2.0 mmol/L (36 mg/dL) transiently, which was reversed immediately with intravenous glucose, resulting in a mean rebound blood glucose of 12.2 ± 2.0 mmol/L (195 ± 3 mg/dL) at 1 h in T2D subjects post-hypoglycemia but remained within the normal glycemic range in the control subjects. Blood sampling was undertaken at baseline, at normoglycemia (5 mmol/L (90 mg/dL)) in T2D subjects, at the point of hypoglycemia, and at 1 h post-hypoglycemia. All subjects developed hypoglycemic symptoms that were immediately reversed (Ethical approval by the NorthWest-Greater Manchester Research Ethics Committee, trial registration NCT03102801, and performed from March 2017–January 2018). Both the mild-hypo and severe-hypo studies were undertaken in the Diabetes Centre at Hull Royal Infirmary. Written informed consent was provided by all participants.

T2D patients had a diabetes duration <10 years with stable hypertensive, lipid-lowering, and diabetes medication (only metformin as an antidiabetic medication was allowed for study inclusion) for at least 3 months prior; HbA1c levels <10% (86 mmol/mol); and no hypoglycemic unawareness or hypoglycemia history during the prior 3-month period. Age was the only parameter that differed between the two groups, with those in the severe-hypo study being older (Table 1).

For control subjects, diabetes was excluded in all with an oral glucose tolerance test. All subjects had normal renal and hepatic function as assessed by biochemical indices, no history of cancer, or any contraindication to hypoglycemia induction with insulin infusion. Medical history, clinical examination, routine blood tests, and an electrocardiogram were performed on all participants.

For the biochemical markers, blood samples were prepared as previously described [12,20]. Blood samples were separated immediately by centrifugation at 2000× *g* for 15 min at 4 °C, and the aliquots were stored at –80 °C within 30 min of blood collection, until batch analysis. High sensitivity C-reactive protein (hsCRP) was measured using a Synchron systems CRPH reagent kit (Beckman-Coulter, High Wycombe, UK) as per manufacturer’s protocol. Fasting plasma glucose (FPG) was measured using a Synchron LX 20 analyzer (Beckman-Coulter) according to the manufacturer’s recommended protocol. Total cholesterol, triglycerides, and high-density lipoprotein (HDL) cholesterol levels were measured enzymatically using a Synchron LX20 analyzer (Beckman-Coulter, High Wycombe, UK).

A SOMAscan assay was used to measure 54 of the 92 cardiovascular protein biomarkers previously described [19], which are the biomarkers available on the SOMAscan panel. These reflected biomarkers are involved in inflammation, cellular metabolic processes, cell adhesion, and immune response and complement activation. As previously described [20], the SOMAscan assay was used to quantify proteins utilizing buffers and SOMAmers from the SOMAscan HTS Assay 1.3 K plasma kit (SomaLogic, Boulder, CO, USA) according to manufacturer’s instructions [21,22]. Initial relative fluorescent units (RFUs) were obtained from microarray intensity images, normalized, and calibrated using the software pipeline provided by SomaLogic. Statistical analyses were performed on log_2_ RFU values using R version 3.5.2 (R Foundation for Statistical Computing, Vienna, Austria), and differential protein expression was analyzed using autonomics and limma [23]. Limma-obtained *p*-values were corrected using the Benjamini–Hochberg method [24].

As this was an explorative study, no calculation of sample size was performed. Data trends were visually evaluated for each parameter and non-parametric tests were applied to data that violated the assumptions of normality when tested using the Kolmogorov–Smirnov test. Comparison between groups was performed at each timepoint using Student’s t-test and multiple comparisons were corrected using the false discovery rate. Within-group comparisons of changes between timepoints were compared using Student’s t-test. Statistical analysis was undertaken using GraphPad Prism (Version 10.2.0, San Diego, CA, USA).

## 3. Results

Age was the only parameter that differed between the two groups for both T2D and controls (Table 1). The results of the cardiovascular risk markers for mild-hypo and severe-hypo are shown in Table 2, and a full list of all markers is shown in Supplementary Table S1.

The markers bone morphogenetic protein 6 (BMP6); signaling lymphocyte activation molecular family 7 (SLAMF7, also known as surface antigen CD319); a thrombospondin type 1 motif, member 13 (ADAMTS13, also known as von Willebrand factor-cleaving protease); and interleukin 1 receptor antagonist (IL1RA), shown to differ in a prior hyperglycemia study [19], were unaffected at the point of induced hypoglycemia in both the hypoglycemia studies, with no additional protein biomarker changes with the rebound hyperglycemia from 1.8 ± 0.1 to 12.2 ± 2.0 mmol/L. 

In the mild-hypo study, T2D subjects showed an increase in Brother of CDO (BOC) (*p* < 0.001), as was also the case in the severe-hypo study (*p* < 0.01) [19] (Table 2).

In the severe-hypo study, baseline to euglycemia showed no change in any of the proteins measured in the T2D cohort. With severe hypoglycemia, study controls showed an increase in Angiopoietin 1 (ANGPT1) (*p* < 0.01) and Dickkopf-1 (DKK1) (*p* < 0.01), but no changes were seen in controls with mild hypoglycemia. In both the mild and severe hypoglycemia studies, at the point of hypoglycemia, T2D showed a decrease in Brother of CDO (BOC) (*p* < 0.01) (Table 1). At one hour after hypoglycemia, the changes in ANGPT1, DKK1, and BOC had resolved, with only one protein, serine protease 27 (PRSS27), becoming elevated in T2D but not in controls, though this was not significant after FDR.

## 4. Discussion

This study suggests that any changes in proteomic cardiovascular risk biomarkers were due to hypoglycemia rather than to fluctuations in glycemic levels per se (as determined by the rebound hyperglycemia following the reversal of the transient severe hypoglycemia in study 2). This study also highlights that any potential increased risk of cardiovascular events due to hypoglycemia is not likely due to changes in the cardiovascular risk markers, as an extensive panel of cardiovascular risk biomarkers was utilized, including those involved in different biological processes which play a role in cardiovascular disease, such as inflammation, cellular metabolic processes, cell adhesion, and immune response and complement activation [19]. This adds further weight to the evidence suggesting that the effect of hypoglycemia on cardiovascular events is through platelet dysfunction via platelet hyperactivity caused by sensitivity to prostacyclin 24 h following the hypoglycemic insult [7] and activation of the sympathetic nervous system leading to hypoglycemia-induced platelet activation [8,9] mediated by an elevation in adrenaline levels [10,11]. In both hypoglycemia studies, only BOC was suppressed at hypoglycemia in T2D, indicating that this protein is likely responsive to hypoglycemia irrespective of its severity though, notably, no change in this protein level was seen in control subjects. BOC is a cell surface receptor that derives its name from the structurally related protein and is related to cell adhesion molecules, is down-regulated by oncogenes, and binds to three Hedgehog ligands [25]. Whilst BOC is not well recognized in diabetes, Hedgehog signaling plays a role in lipid metabolism, insulin sensitivity, inflammatory response, and diabetes-related complications [26], and its deficiency in animal models is associated with the development of neuropathy [27], thus linking it with diabetes-related complication pathogenesis.

This study also suggests that the cardiovascular risk markers BMP6, SLAMF7, ADAMTS13, and IL1RA, shown previously by others to respond to transient elevations in glucose [19], do not respond to a fall in glucose or rebound hyperglycemia in T2D. It is recognized that hyperglycemia is associated with diabetes-related complications and the reduction in mean blood glucose reduces their incidence [15]. This suggests that in the absence of cardiovascular risk protein marker changes consequent upon the reduction in blood glucose, the association of glucose variability with complications may be entirely due to the mean time within hyperglycemia of those glucose excursions, rather than the glucose fluctuations per se.

The strengths of this study include inclusion of T2D subjects having a relatively short disease duration and not on polypharmacy (with no difference in these parameters between studies) and that the same hyperinsulinemic clamp protocol for hypoglycemia was utilized in both studies. Limitations of this study include that the panel previously reported utilized Olink proteomic measurements [19], so it was not identical to the SOMAscan panel; this resulted in the ILRA4, LOX-1, and PTX3 proteins not being included in the SOMAscan panel, so no conclusions can be drawn about them. The relatively small subject numbers in each study cohort, for both T2D and control subjects, is a limitation; however, whilst larger subject numbers may have revealed changes in plasma proteins, there were no trends to suggest that this may be the case. As subjects enrolled in these studies were all Caucasian, these results may not be generalizable to other ethnic populations.

In conclusion, proteomic biomarkers of cardiovascular disease showed changes at hypoglycemia that resolved within 1 h after the hypoglycemic event and with no changes following hyperglycemia rebound, suggesting that any cardiovascular risk increase is due to hypoglycemia and not to glucose fluctuation per se.

## Figures and Tables

**Table 1:** Demographic and biochemical parameters of control (Ctrl) and type 2 diabetic (T2D) subjects included in study 1 (mild hypoglycemia) and study 2 (severe hypoglycemia). Data are presented as mean ± SD.

	Study 1 Ctrl (*n* = 7)	Study 2 Ctrl (*n* = 23)	*p*-Value	Study 1 T2D (*n* = 10)	Study 2 T2D (*n* = 23)	*p*-Value
Age (years)	47 ± 6	60 ± 10	0.003	46 ± 6	64 ± 8	<0.0001
Sex (M/F)	4M/3F	11M/12F		7M/3F	12M/11F	
BMI (kg/m^2^)	29 ± 4	28 ± 3	0.640	36 ± 7	32 ± 4	0.03
Systolic BP (mmHg)	126 ± 15	122 ± 8	0.280	127 ± 20	132 ± 8	0.31
Diastolic BP (mmHg)	75 ± 13	75 ± 6	1.000	75 ± 11	81 ± 7	0.08
Duration of diabetes (years)	N/A	N/A		3.3 ± 2.3	4.5 ± 2.2	0.14
HbA1c (mmol/mol)	33.6 ± 2.9	37.2 ± 2.2	0.004	49 ± 12	51 ± 11	0.62
HbA1c (%)	5.2 ± 0.3	5.6 ± 0.2	0.006	6.6 ± 1.0	6.8 ± 1.0	0.48
Total cholesterol (mmol/L)	5.1 ± 0.8	4.8 ± 0.77	0.230	5.3 ± 0.7	4.2 ± 1.0	0.36
Triglyceride (mmol/L)	1.2 ± 0.5	1.3 ± 0.6	0.540	1.7 ± 0.8	1.7 ± 0.7	0.96
CRP (mg/L)	0.8 ± 0.0	5.1 ± 10.3	0.26	2.8 ± 1.8	3.1 ± 2.9	0.94

BMI—body mass index; BP—blood pressure; HbA1c—glycated hemoglobin; CRP—C-reactive protein; N/A—Not applicable.

**Table 2:** Protein levels at baseline (BL) and hypoglycemia (Hypo) reported as mean ± standard deviation (SD) of relative fluorescent units (RFU) as measured by SOMAscan assay.

Protein	Study1—Ctrl		Study2—Ctrl		Study1—T2D		Study2—T2D	
	Mean **±** SD BL vs. Hypo	*p*-Value	Mean **±** SD BL vs. Hypo	*p*-Value	Mean **±** SD BL vs. Hypo	*p*-Value	Mean **±** SD BL vs. Hypo	*p*-Value
BMP6	BL: 1916 **±** 507 Hypo: 2006 **±** 205	0.69	BL: 14187 **±** 4475 Hypo: 13753 **±** 4286	0.73	BL: 5034 **±** 9854 Hypo: 5464 **±** 9438	0.92	BL: 13729 **±** 5119 Hypo: 13054 **±** 5144	0.65
SLAMF7	BL: 58124 **±** 20707 Hypo: 54791 **±** 12294	0.73	BL: 41917 **±** 14548 Hypo: 38021 **±** 13984	0.35	BL: 73898 **±** 26200 Hypo: 70961 **±** 27635	0.81	BL: 44153 **±** 20302 Hypo: 37646 **±** 17153	0.24
ADAMTS13	BL: 4500 **±** 1065 Hypo: 4988 **±** 1230	0.98	BL: 3921 **±** 845 Hypo: 3949 **±** 1049	0.92	BL: 5231 **±** 1164 Hypo: 5194 **±** 1047	0.94	BL: 4080 **±** 1062 Hypo: 4118 **±** 893	0.89
IL1RA	NA		BL: 5386 **±** 3101 Hypo: 5261 **±** 3012	0.89	NA		BL: 4971 **±** 2477 Hypo: 4490 **±** 2118	0.47
BOC	BL: 1541 **±** 359 Hypo: 1263 **±** 199	0.12	BL: 1618 **±** 489 Hypo: 1489 **±** 448	0.35	BL: 1565 **±** 343 Hypo: 992 **±** 311	0.001	BL: 1475.8 **±** 355 Hypo: 1216 **±** 309	0.01
ANGPT1	BL: 942 **±** 494 Hypo: 646 **±** 99	0.17	BL: 433 **±** 156 Hypo: 815 **±** 667	0.01	BL: 766 **±** 209 Hypo: 932 **±** 598	0.42	BL: 752 **±** 610 Hypo: 1007.7 **±** 695.0	0.18
DKK1	BL: 15699 **±** 6353 Hypo: 11425 **±** 3511	0.17	BL: 18152 **±** 8054 Hypo: 27728 **±** 16313	0.02	BL: 14757 **±** 2338 Hypo: 17166 **±** 13612	0.59	BL: 28249 **±** 17077 Hypo: 34616 **±** 16789	0.20

BMP6—bone morphogenetic protein 6; SLAMF7—signaling lymphocytic activation molecule (SLAM) family member 7; ADAMTS13—a disintegrin and metalloproteinase with thrombospondin motif 13; IL1RA—interleukin-1 receptor antagonist protein; BOC—Brother of CDO; ANGPT1—Angiopoietin-1; DKK1—Dickkopf-related protein 1.

## Data Availability

All the data for this study will be made available upon reasonable request to the corresponding author.

## Supplementary Materials

The following supporting information can be downloaded at: https://www.mdpi.com/article/10.3390/biomedicines12061137/s1, Table S1: *p* values for all Protein levels at baseline (BL) vs hypoglycemia (Hypo) in T2D and Control in Study1 and Study2.
