# Supplementary material for: Effect of Hypoglycemia and Rebound Hyperglycemia on Proteomic Cardiovascular Risk Biomarkers
Source: Biomedicines. 2024 May 21;12(6):1137. doi: 10.3390/biomedicines12061137 (PMC11201283; doi:10.3390/biomedicines12061137)
Supplement: Supplementary file 1 [file biomedicines-12-01137-s001.zip › biomedicines-2998260-supplementary.pdf]

					STUDY1										STUDY2					
PROTEINS			T2D					CONTROL					T2D					CONTROL		
	BL-Mean	BL-SD	Hypo-Mean	Hypo-SD	p-value	BL-MEAN	BL-SD	Hypo-Mean	Hypo-SD	p-Value	BL-MEAN	BL-SD	Hypo-Mean	Hypo-SD	p-Value	BL-Mean	BL-SD	Hypo-Mean	Hypo-SD	p-value
BMP6	5034.35	9853.79	5464.15	9437.84	0.92	1916.14	507.21	2005.92	205.02	0.69	13728.84	5118.92	13053.94	5143.75	0.65	14187.35	4475.13	13753.38	4285.81	0.74
SLAMF7	73898.27	26199.67	70961.08	27634.94	0.81	58124.33	20707.44	54791.35	12293.90	0.74	44153.42	20301.79	37646.23	17153.16	0.24	41917.20	14547.73	38020.59	13984.19	0.35
ADAMTS13	5231.47	1164.37	5194.25	1046.85	0.94	4999.86	1065.62	4987.98	1229.55	0.99	4079.73	1062.04	4118.08	893.31	0.89	3921.22	844.86	3948.80	1048.70	0.92
IL1RN	...										4970.82	2476.97	4489.53	2118.34	0.48	5386.29	3100.68	5261.02	3012.00	0.89
ANGPT1	766.26	208.83	931.77	597.46	0.42	942.36	494.01	646.43	98.88	0.18	751.67	609.50	1007.75	695.01	0.19	432.58	155.55	815.36	667.21	0.01
ADM	...										1577.02	225.32	1506.31	149.49	0.21	1569.97	157.99	1541.36	195.98	0.59
PGF	604.77	281.46	529.72	72.33	0.42	515.41	85.09	697.32	373.19	0.23	299.11	46.46	321.31	55.74	0.15	370.33	287.09	382.23	310.58	0.89
ADAMTS13	5231.47	1164.37	5194.25	1046.85	0.94	4999.86	1065.62	4987.98	1229.55	0.99	4079.73	1062.04	4118.08	893.31	0.89	3921.22	844.86	3948.80	1048.70	0.92
BOC	1564.82	343.36	992.35	311.27	0.00	1541.33	359.34	1262.97	199.59	0.12	1475.82	355.03	1215.64	FALSE	0.01	1617.80	488.73	1488.96	448.25	0.35
SRC	14024.99	7753.02	11670.94	5466.43	0.44	11035.69	4661.24	10548.72	6295.65	0.88	7058.97	3825.78	6487.60	3565.28	0.60	5609.77	3140.43	4854.73	3119.58	0.41
IL6	424.50	320.34	506.78	315.45	0.57	301.14	109.65	413.53	92.38	0.07	192.14	27.87	196.84	25.81	0.55	208.40	82.70	233.03	115.39	0.41
TNFRSF10A	2165.93	3640.48	2048.33	3535.36	0.94	1488.50	2040.04	1963.12	1985.57	0.68	639.33	709.69	625.42	511.98	0.94	517.67	152.68	558.43	139.92	0.34
IDUA	1405.98	604.51	1220.57	345.38	0.41	1409.41	437.86	1226.95	632.40	0.55	1607.88	692.47	1626.80	644.79	0.92	1114.97	308.26	1201.29	456.20	0.45
TNFRSF11A	973.83	271.68	1409.49	597.24	0.05	1191.71	609.29	1265.45	654.75	0.84	513.03	82.22	507.95	83.59	0.83	556.67	71.49	600.99	326.11	0.53
TNFRSF10B	...										260.48	62.10	294.92	76.48	0.10	255.19	57.79	266.12	81.80	0.60
PRSS27	5799.48	1986.70	7201.24	2282.24	0.16	4994.54	1017.50	5188.72	1293.82	0.77	1729.97	187.18	1798.53	232.27	0.27	1864.93	291.28	1843.60	334.96	0.82
TEK	2054.47	220.94	1980.28	202.70	0.44	2120.46	231.16	2061.90	315.61	0.71	1524.60	349.17	1448.10	367.73	0.47	1660.93	337.18	1527.36	337.82	0.18
F3	1778.00	1886.31	953.83	174.39	0.19	1113.19	209.55	2304.57	2540.07	0.24	1617.43	461.75	1478.40	450.38	0.30	1652.26	1153.83	1612.32	1053.31	0.90
PDGFRB	6095.67	2036.41	5009.11	2164.28	0.26	7881.97	2196.71	6551.32	2020.43	0.28	6957.87	2796.57	6841.73	2716.83	0.89	7140.82	3299.42	7285.60	2985.66	0.88
IL27	1095.64	394.63	939.27	158.20	0.26	883.26	138.69	992.55	266.54	0.36	1142.82	2660.60	1070.94	2413.74	0.92	753.61	619.75	762.67	678.53	0.96
CXCL1	3035.41	768.61	4490.60	3156.86	0.17	3068.44	1321.76	2189.08	270.28	0.14	2487.27	848.28	2699.48	774.04	0.37	2060.34	319.85	2288.43	684.25	0.15
LGALS9	...										1567.87	1092.77	1326.33	570.87	0.34	1531.41	602.73	1533.13	821.91	0.99
IL18R1	14166.11	3947.97	13347.56	2217.14	0.57	11979.61	2871.31	10761.47	2577.72	0.44	9761.40	2171.63	9276.38	2536.58	0.49	9509.92	2968.69	8627.93	2604.45	0.28
PIGR	5733.85	2476.00	5570.18	1832.12	0.87	4726.03	864.12	3850.72	752.27	0.08	3747.57	1645.52	3415.77	1280.93	0.44	2940.66	1001.80	2903.82	1026.29	0.90
AGER	647.41	221.46	525.68	215.32	0.23	605.37	237.80	614.48	191.07	0.94	460.83	263.92	413.86	227.81	0.52	617.58	284.46	603.25	222.33	0.85
SOD2	49390.12	12748.29	45925.91	9114.18	0.49	47948.27	11609.44	52184.53	6967.08	0.45	36630.83	7731.56	37736.25	11696.33	0.71	50276.75	21710.84	51404.37	23294.57	0.86
GH1	...										284.46	134.45	304.01	133.17	0.62	254.40	160.88	271.16	146.63	0.71
FST	750.61	83.69	874.19	213.44	0.11	709.56	58.64	676.18	40.99	0.27	558.25	43.08	547.01	52.66	0.43	549.69	31.49	530.15	41.67	0.08
CD84	6551.31	393.95	6363.17	576.96	0.41	6228.93	1276.47	6892.03	389.15	0.25	4472.00	1032.49	4121.27	983.01	0.24	4829.14	1026.86	5078.98	1089.15	0.42
PAPPA	12345.27	3538.76	13738.59	5002.03	0.48	10848.79	4471.38	17307.50	4555.47	0.03	15260.25	4664.56	16840.77	5527.61	0.30	15070.80	5577.72	16248.36	5034.81	0.45
REN	726.09	212.37	777.12	219.67	0.60	921.97	372.19	777.18	254.18	0.44	1729.84	2713.65	1601.35	2503.66	0.87	674.73	344.59	665.84	344.18	0.93
THBS2	18746.86	5151.37	14958.30	2757.25	0.06	14174.03	6071.29	11923.32	4172.65	0.46	14211.00	4945.19	11987.96	3582.72	0.08	12746.65	5865.42	11158.08	4888.20	0.32
XCL1	340.43	27.59	316.88	23.33	0.05	321.24	22.33	346.08	27.20	0.10	380.22	98.67	395.53	89.16	0.58	344.66	43.85	367.51	58.44	0.14
CCL13	311.21	19.83	353.96	50.60	0.02	474.54	244.44	462.60	296.92	0.94	257.19	45.98	272.03	52.31	0.31	242.18	24.08	265.59	106.60	0.31
IL16	1447.34	345.63	1346.13	431.87	0.57	1524.41	467.50	1609.78	302.14	0.71	193.16	73.11	191.45	44.02	0.92	175.67	30.70	169.90	28.48	0.51
CCL17	810.72	143.86	764.30	66.38	0.37	966.20	543.67	702.80	56.25	0.27	1131.27	767.39	1296.50	910.18	0.51	756.40	317.83	995.11	603.27	0.10
CCL3	1492.03	336.41	1285.74	338.62	0.19	1166.14	313.20	1295.27	381.45	0.52	633.60	202.11	638.70	199.16	0.93	713.23	290.05	701.06	342.89	0.90
MMP7	955.59	183.35	886.58	125.93	0.34	852.57	113.11	873.10	150.54	0.78	1241.52	447.96	1175.76	306.87	0.56	1004.58	200.53	968.72	248.37	0.59
FCGR2A FCGR2I	2981.50	2100.78	2313.29	1626.18	0.44	2530.13	885.65	3375.52	1462.49	0.23	1959.41	1310.66	1739.20	1332.98	0.57	2270.49	1528.07	2024.83	1207.03	0.54
DCN	2464.78	312.32	2401.08	461.78	0.72	2707.31	302.88	2284.25	285.49	0.03	4074.90	736.49	4222.91	778.59	0.51	3709.53	690.11	3929.01	777.52	0.31
DKK1	14757.16	2338.18	17165.60	13611.51	0.59	15698.84	6353.02	11425.37	3511.40	0.17	28249.02	17076.66	34615.85	16788.68	0.20	18151.63	8053.68	27728.12	16312.60	0.01
AGRP	1214.75	262.54	1023.22	306.49	0.15	1218.67	258.37	1565.95	249.58	0.03	1332.24	608.77	1246.53	578.85	0.62	1313.85	267.26	1370.07	318.67	0.52
HBEGF	200.87	36.74	233.92	67.74	0.19	218.87	36.82	203.40	24.06	0.40	109.60	14.57	109.78	15.50	0.97	114.27	40.17	111.42	34.03	0.79
GDF2	1505.93	250.98	1415.23	210.81	0.39	1309.07	244.21	1378.55	221.22	0.60	1794.83	442.86	1799.86	475.56	0.97	1784.26	410.29	1775.70	362.12	0.94
THPO	...										134.16	20.25	138.74	23.72	0.48	138.42	39.23	151.81	38.51	0.24
MMP12	753.26	115.42	618.67	125.55	0.02	637.10	108.93	626.27	103.74	0.86	1450.95	992.68	1238.90	896.53	0.45	1072.20	395.95	979.61	446.35	0.46
ACE2	...										291.61	146.80	286.32	139.97	0.90	281.23	86.90	276.97	82.85	0.86
PDCD1LG2	4324.95	1724.31	3496.71	756.46	0.18	4560.29	718.95	4688.60	2132.78	0.88	3783.55	946.15	3459.96	1001.87	0.26	3805.53	992.82	3795.07	1102.02	0.97
TNFRSF13B	921.03	110.50	1562.78	1784.95	0.27	1698.04	2075.81	939.88	96.41	0.39	1358.22	192.82	1375.06	174.02	0.75	1464.58	253.44	1347.48	265.96	0.13
LEP	12043.00	10421.09	9728.37	6827.76	0.56	10074.51	7375.65	9830.50	9302.17	0.96	12917.54	8710.13	11375.78	7842.90	0.53	13731.94	9203.88	14328.38	9958.51	0.83
CD4	331.43	27.95	328.73	24.38	0.82	334.23	18.10	362.27	31.05	0.07	283.03	30.51	289.97	33.97	0.47	259.33	19.46	275.15	32.99	0.05
IGHE IGK@ IGL(	5194.74	8162.04	6114.72	8494.40	0.81	4940.26	5661.66	4048.47	4255.89	0.76	3120.12	2402.63	2977.73	2436.49	0.84	4082.53	7073.56	4471.69	7639.30	0.86
FGF23	1308.48	1475.12	955.90	1315.39	0.58	391.57	62.52	802.30	827.85	0.21	420.07	50.73	414.60	49.51	0.71	544.85	299.90	546.77	236.18	0.98
NPPB	653.01	665.93	582.79	537.33	0.80	375.26	107.07	502.18	410.82	0.45	387.95	86.64	412.30	88.00	0.34	380.79	85.11	408.01	88.08	0.29
IL2RA	1747.21	1300.60	1666.08	785.12	0.87	1069.09	269.11	1753.62	1577.13	0.28										
